# Mammographic compression: a force to be reckoned with

**DOI:** 10.1038/sj.bjc.6690863

**Published:** 1999-12

**Authors:** G Stehle1, A Wunder, G Hartung, H Sinn, D L Heene

**Affiliations:** I. Medical Clinic andII Medical Clinic, University Clinic Mannheim, University of Heidelberg, D-68135 Mannheim,Germany; Department of Radiochemistry and Radiopharmacology E 0300, German Cancer Research Center, 69120 Heidelberg, Germany


*British Journal of Cancer* (1999) **81**(8), 1426–1428
© 1999 Cancer Research Campaign
Article no. bjoc.1999.0862

### Mammographic compression: a force to be reckoned with

**Sir**,

Thornton and Baum (1999) advocate the establishment of a Citizen's Jury to provide an open and enlightened forum for discussing the introduction of a mammography screening programme. Yet one essential witness to their jury appears to be missing – an investigator who looks at this potentially painful and injurious procedure from a biological perspective.

Pain is a physiological warning sign for injury. In response to injury, serum or wound fluid is formed to stimulate tissue repair. If the wound is sterile, immune cytotoxic responses are not required and only cellular regeneration is necessary. Thus, the mitogenic properties of serum make it an excellent growth medium for tumour cells. However, serum-stimulated tumour cell growth is not simply an in vitro phenomenon, but occurs in vivo as well – as demonstrated in a recent article in this journal (Abramovitch et al, 1999). The investigators examined the effect of injected wound fluid on the growth and vascularization of implanted tumour spheroids in nude mice. They found that wound fluid from sites of injury was directly mitogenic to tumour cells and led to accelerated tumour growth, and that such fluid may trigger 'the angiogenic switch of avascular, dormant microtumours'. In humans, an association between physical injury and the subsequent development/progression of breast and other cancers has long been suspected (Coley, 1911).

We are concerned that the potential compressive injury resulting from mammography could augment the growth dynamics of dormant or slow growing tumours (van Netten et al, 1994). The 'discomfort' induced by mammographic compression is not trivial (Poulos and Rickard, 1997), nor is the more vigorous compressive follow-up performed on women with suspicious mammograms (van Netten et al, 1997). Bruising is not an uncommon result of this procedure (Elwood et al, 1998). Investigators should consider that the physical pain and injury are more than just deterrents to further screening. During compression (up to 200 N or 45 lbs), the less dense fatty tissue will easily redistribute, however, this leaves the brunt of compressive force on the denser tumour tissue.

Thus a procedure, which has both potential for benefit and harm, may explain the negative findings of the recently published analysis of the Swedish mammography programme (Sjonell and Stahle, 1999). At the 10-year review of a programme involving over 600 000 participants, no significant mortality reduction in women 50–69 was observed. In theory, one would expect that the removal of in situ and other early stage carcinomas in a significant proportion of women would reduce mortality from this disease – unless, simultaneously, this compressive procedure could alter the nature of this disease by enhancing its spread and increasing its rate of growth. Women who experience intense pain during this procedure may be most at risk and alternatives to compressive screening are needed. We predict that such a screening programme will have a significant effect on mortality reduction. Furthermore, modern epidemiological studies examining the association between injury and subsequent malignant progression are required to elucidate how trauma may act as a co-carcinogen.

*JP van Netten,*[1,2] *SA Cann*[1,2] *and DW Glover*[1]
[1]*Special Development Laboratory, Royal Jubilee Hospital, Capital Health Region, Victoria, BC, V8R 1J8, Canada;*
[2]*Department of Biology, University of Victoria, Victoria, BC V8W 3N5, Canada*

### REFERENCES

Abramovitch R, Marikovsky M, Meir G and Neeman M (1999) Stimulation of tumour growth by wound-derived growth factors. *Br J Cancer* **79**: 1392–1398
Coley WB (1911) Injury as a causative factor in cancer. *Ann Surg* **4**: 449–482
Elwood M, McNoe B, Smith T, Bandaranayake M and Doyle TCA (1998) Once is enough: why some women do not continue to participate in a breast cancer screening programme. *N Z Med J* **111**: 180–183
Poulos A and Rickard M (1997) Compression mammography and the perception of discomfort. *Australas Radiol* **41**: 247–252
Thornton H and Baum M (1999) Should a mammographic screening programme carry the warning: screening can damage your health? *Br J Cancer* **79**: 691–692
Sjonell G and Stahle L (1999) Mammographic screening does not reduce breast cancer mortality. *Lakartidningen* **96**: 904–905, 908–913
van Netten JP, Mogentale T, Ashwood-Smith MJ, Fletcher C and Coy P (1994) Physical trauma and breast cancer. *Lancet* **343**: 978–979
van Netten JP, Cann SA and Hall JG (1997) Mammography controversies: time for informed consent? *J Natl Cancer Inst* **89**: 1164–1165

### Mammographic compression: a force to be reckoned with—reply

**Sir**,

Those who organize a Citizens' Jury have considerable power and responsibility. Not only are they required to select the witnesses but they have to brief the jury, organize the presentation of the information, moderate on the proceedings and report on the findings.

It is thus important that organizers commissioned to arrange this democratic way of examining important questions enabling people to exercise their citizenship, recognize their accountability both to the sponsors and the public. They must be open and fair in all the aspects listed above. Consideration of selection of witnesses, both those who volunteer and those to be invited, must allow for as wide and balanced a spectrum of considerations as possible so that a representative cross-section of opinion is provided. This presentation of evidence then offers some chance of a fair verdict being decided by 12 (or more!) good men and true.

We are therefore pleased that our suggestion has resulted in the first volunteer. Our list of witnesses was not intended to be exhaustive, final or complete: only to stimulate consideration of this democratic method and elicit constructive suggestions.

*H Thornton and M Baum*

### Prognostic value of cathepsin D in breast cancer

**Sir**

A recent publication in this journal demonstrated, that cathepsin D was an independent marker of poor prognosis in 2810 breast cancer patients (Foekens et al, 1999). A covering editorial rose questions on the still unexplained biological role of cathepsin D in breast cancer (Westley et al, 1999). Cathepsin D levels correlated with increasing invasiveness and high metastatic potential of tumour cells in breast cancer. Breast cancer cells were found to secrete cathepsin D into the cell culture medium. Thus, it is commonly hypothesized, that cathepsin D might be involved in the extracellular breakdown of the tumour stroma in vivo and contribute to tumour dissemination. This hypothesis needs comment and should be modified in view of recent findings. High cathepsin D levels are not only present in breast cancer samples, but have been described for a multitude of other tumour entities, such as laryngeal cancer, cancers of the head and neck, ovarian cancer, endometrial cancer, bladder cancer, or in malignant glioma. In these reports, high cathepsin D levels were usually also considered as a sign of a poor prognosis. High levels of other lysosomal cathepsins B, H and L have been detected in a variety of different cancers including breast cancer, as well (Recklies et al, 1980). These observations clearly indicate that a highly active lysosomal system is characteristic of cancer tissue.

Lysosomes are involved in the degradation of extracellular material ingested by endocytosis. Cathepsins are intralysosomally active at pH 3–5 and loose most activity at a neutral pH (Bohley et al, 1992). Although cathepsin D might be secreted by cancer cells, there is no evidence available that the secreted enzyme is actively involved in the extracellular degradation of the extracellular matrix (ECM) at the neutral or slightly acidic pH in tumour tissues in vivo. In cell culture, extracellular degradation of ECM mediated by cathepsin D was only achieved after lowering the pH to 4.5 in the cell culture media, but not at pH 7.4 (Briozzo et al, 1988). When invasiveness of breast cancer cells was studied in the Boyden chamber, the secretion of cathepsin D even correlated inversely with the aggressive behaviour of the cancer cells (Johnson et al, 1993). To our knowledge extracellular acidic compartments containing proteases of lysosomal origin involved in a break-down of ECM, have not been found in tumour tissues.

In contrast, there are a variety of other formidable proteases available, which are responsible for extracellular proteolysis of the ECM (recently reviewed by Price et al, 1997). All of these are active at a neutral pH, and highly regulated by their respective inhibitors. These proteases belong to the plasminogen activator (PA) or to the matrix metalloproteinase (MMP) superfamilies. Increased expression of PA and MMPs in a variety of tumours, including breast cancer, are associated with a high metastatic potential and poor prognosis (Stetler Stevenson et al, 1996; Duffy et al, 1998). Invasive cells form specialized membrane protrusions, called invadopodia, that actively degrade the surrounding ECM (Kelly et al, 1994). MMPs are structurally and functionally linked with invadopodia (Chen, 1996; Nakahara et al, 1997). To our knowledge there are no reports on cathepsin activity in connection to invadopodia so far.

Instead, considerable evidence points to an important role for cathepsins as proteolytic enzymes in the lysosomal system. Invasive breast cancer cells were able to ingest considerable amounts of extracellular material in large acid phagosomes (pH < 4). The presence of these phagosomes coincided with an increased ability of these cells to migrate through matrigel, and with high cathepsin D levels (Montcourrier et al, 1990, 1994). Recent data support these observations, indicating that MMPs were responsible for the partial degradation of a cross-linked gelatine matrix. The gelatin fragments were taken up by phagocytosis and destined to lysosomes for further degradation. This phagocytic capacity of breast cancer cells was directly linked to their invasiveness (Coopman et al, 1998).

Further observations strengthen the concept of an active lysosomal digestive system and hence high cathepsin D levels in malignant tumours. Tumours have been termed nitrogen traps. During tumour cell proliferation extensive amounts of nitrogenous substrates are required to provide material for cell duplication (new tumour proteins, DNA, RNA …). Free amino acid concentrations in blood are low, leaving only plasma proteins (mostly albumin) as the major external source for additional nitrogen, necessary during tumour proliferation (Stehle et al, 1997). And indeed, there are reports confirming albumin accumulation and degradation in tumours (Sinn et al, 1990; Andersson et al, 1991; Wunder et al, 1997). In sarcoma-bearing mice the intralysosomal accumulation of residualizingly radiolabelled albumin was threefold higher in tumour tissue compared to liver tissue. Accounting for the entire tumour the proteolytic capacity for albumin breakdown exceeded that of the healthy liver by a factor of five (Andersson et al, 1991). Interestingly, cathepsin D is closely involved in the lysosomal catabolism of albumin (Mego, 1984). A considerably increased intracellular albumin content was found in an immunohistological study of breast cancer tissue (Vercelli Retta et al, 1987). High plasma protein (albumin) accumulation was also detected in homogenized tumour tissue from mastectomy specimens. A significant correlation between high plasma protein content in the samples and poor prognosis in breast cancer patients was established (Soreide et al, 1991*a*, 1991*b*).

ECM fragments and plasma proteins have been identified as substrates for lysosomal cathepsins. Tumour cell apoptosis might

© 1999 Cancer Research Campaign provide further material doomed for lysosomal digestion. Tumours have a high rate of cell turnover, and e.g. the cell loss was established as high as 96% (Refsum et al, 1967). Remnants of apoptotic cells might be recognized and rapidly phagocytosed by neighbouring cells in the tissue (Collins et al, 1993). A high apoptotic index in breast cancer cells was associated with a high mitotic rate, tumour necrosis, dense stromal lymphocyte infiltration, lack of tubule formation, lack of sex steroid receptor expression, a high grade tumour and with a short recurrence free survival (Lipponen et al, 1994). In addition, macrophages might also be involved in the phagocytosis of apoptotic cells (Savill et al, 1993). The role of macrophages, leucocytes, fibroblasts or of neighbouring tumour cells in the clearance and degradation of apoptotic cell remnants still remain to be further clarified. Interestingly, high cathepsin D levels also coincide with all of these cell types present in breast cancer tissue.

Based on these observations, we conclude that the biological role of cathepsin D is mostly confined to the lysosomal system. High cathepsin D levels in tumour tissues reflect high lysosomal turnover. This turnover is likely to be caused by an increased uptake and digestion of ECM fragments, of plasma proteins, and of cellular debris (remnants of apoptotic cells?) mostly by proliferating cancer cells. The ingested and degraded materials might serve as tumour cell nutrients. A positive correlation of cathepsin D with tumour malignancy or metastatic potential is obvious. It is evident, that further research is needed to investigate the role of the lysosomal system during tumour cell proliferation.

*G Stehle[1], A Wunder[3], G Hartung[2], H Sinn[3] and DL Heene[1]*
*[1]I. Medical Clinic and [2]II Medical Clinic, University Clinic Mannheim, University of Heidelberg, D-68135 Mannheim, Germany; [3]Department of Radiochemistry and Radiopharmacology E 0300, German Cancer Research Center, 69120 Heidelberg, Germany.*

### REFERENCES

Andersson C, Iresjo BM and Lundholm K (1991) Identification of tissue sites for increased albumin degradation in sarcoma-bearing mice. *J Surg Res* **50**: 156–162

Bohley P and Seglen PO (1992) Proteases and proteolysis in the lysosome. *Experientia* **48**: 151–157

Briozzo P, Morisset M, Capony F, Rougeot C and Rochefort H (1988) In vitro degradation of extracellular matrix with *Mr* 52 000 cathepsin D secreted by breast cancer cells. *Cancer Res* **48**: 3688–3692

Chen WT (1996) Proteases associated with invadopodia, and their role in degradation of extracellular matrix. *Enzyme Protein* **49**: 59–71

Collins MK and Lopez Rivas A (1993) The control of apoptosis in mammalian cells. *Trends Biochem Sci* **18**: 307–309

Coopman PJ, Do MT, Thompson EW and Mueller SC (1998) Phagocytosis of cross-linked gelatin matrix by human breast carcinoma cells correlates with their invasive capacity. *Clin Cancer Res* **4**: 507–515

Duffy MJ and McCarthy K (1998) Matrix metalloproteinases in cancer: prognostic markers and targets for therapy (review). *Int J Oncol* **12**: 1343–1348

Foekens JA, Look MP, Bolt-de Vries J, Meijer-van Gelder ME, van Putten WLJ and Klijn JGM (1999) Cathepsin-D in primary breast cancer: prognostic evaluation involving 2810 patients. *Br J Cancer* **79**: 300–307

Johnson MD, Torri JA, Lippman ME and Dickson RB (1993) The role of cathepsin D in the invasiveness of human breast cancer cells. *Cancer Res* **53**: 873–877

Kelly T, Mueller SC, Yeh Y and Chen WT (1994) Invadopodia promote proteolysis of a wide variety of extracellular matrix proteins. *J Cell Physiol* **158**: 299–308

Lipponen P, Aaltomaa S, Kosma VM and Syrjanen K (1994) Apoptosis in breast cancer as related to histopathological characteristics and prognosis. *Eur J Cancer* **30**: 2068–2073

Mego JL (1984) Role of thiols, pH and cathepsin D in the lysosomal catabolism of serum albumin. *Biochem J* **218**: 775–783

Montcourrier P, Mangeat PH, Salazar G, Morisset M, Sahuquet A and Rochefort H (1990) Cathepsin D in breast cancer cells can digest extracellular matrix in large acidic vesicles. *Cancer Res* **50**: 6045–6054

Montcourrier P, Mangeat PH, Valembois C, Salazar G, Sahuquet A, Duperray C and Rochefort H (1994) Characterization of very acidic phagosomes in breast cancer cells and their association with invasion. *J Cell Sci* **107**: 2381–2391

Nakahara H, Howard L, Thompson EW, Sato H, Seiki M, Yeh Y and Chen WT (1997) Transmembrane/cytoplasmic domain-mediated membrane type 1-matrix metalloprotease docking to invadopodia is required for cell invasion. *Proc Natl Acad Sci USA* **94**: 7959–7964

Price JT, Bonovich MT and Kohn EC (1997) The biochemistry of cancer dissemination. *Crit Rev Biochem Mol Biol* **32**: 175–253

Recklies AD, Tiltman KJ, Stoker TA and Poole AR (1980) Secretion of proteinases from malignant and nonmalignant human breast tissue. *Cancer Res* **40**: 550–556

Refsum SB and Berdal P (1967) Cell loss in malignant tumours in man. *Eur J Cancer* **3**: 235–236

Savill J, Fadok V, Henson P and Haslett C (1993) Phagocyte recognition of cells undergoing apoptosis. *Immunol Today* **14**: 131–136

Sinn H, Schrenk HH, Friedrich EA, Schilling U and Maier Borst W (1990) Design of compounds having an enhanced tumour uptake, using serum albumin as a carrier. Part I. *Int J Rad Appl Instrum B* **17**: 819–827

Soreide JA, Lea OA and Kvinnsland S (1991a) Cytosol albumin content in operable breast cancer. Correlations to steroid hormone receptors, other prognostic factors and prognosis. *Acta Oncol* **30**: 797–802

Soreide JA, Lea OA and Kvinnsland S (1991b) Cytosol protein content and prognosis in operable breast cancer. Correlations with steroid hormone receptors and other prognostic factors. *Breast Cancer Res Treat* **20**: 25–32

Stehle G, Sinn H, Wunder A, Schrenk HH, Stewart JCM, Hartung G, Maier-Borst W and Heene DL (1997) Plasma protein (albumin) catabolism by the tumour itself-implications for tumor metabolism and the genesis of cachexia. *Crit Rev Oncol Hematol* **26**: 77–100

Stetler Stevenson WG, Hewitt R and Corcoran M (1996) Matrix metalloproteinases and tumor invasion: from correlation and causality to the clinic. *Semin Cancer Biol* **7**: 147–154

Vercelli Retta J, Manana G, Almeida E, Chiribao C, Estevez A and Moro R (1987) Normal serum proteins in female breast carcinomas and fibroadenomas. An immunohistochemical study. *Ann Pathol* **7**: 209–215

Westley BR and May FEB (1999) Prognostic value of cathepsin D in breast cancer. *Br J Cancer* **79**: 189–190

Wunder A, Stehle G, Sinn H, Schrenk HH, Hoff-Biederbeck D, Bader F, Friedrich EA, Peschke P, Maier-Borst W and Heene DL (1997) Enhanced albumin uptake by rat tumors. *Int J Oncol* **11**: 497–507

*© 1999 Cancer Research Campaign*

